# Transcriptome and Parasitome Analysis of Beet Cyst Nematode *Heterodera schachtii*

**DOI:** 10.1038/s41598-020-60186-0

**Published:** 2020-02-24

**Authors:** Abdelnaser M. Elashry, Samer S. Habash, Paramasivan Vijayapalani, Nahal Brocke-Ahmadinejad, Roman Blümel, Arun Seetharam, Heiko Schoof, Florian M. W. Grundler

**Affiliations:** 1 INRES Molecular Phytomedicine, University of Bonn, Karlrobert-Kreiten-Str. 13, Bonn, 53115 Germany; 2 Department of Plant Pathology and Microbiology, Iowa State University, Ames, IA 50011 USA; 3 INRES Crop Bioinformatics, University of Bonn, Katzenburgweg 2, 53115 Bonn, Germany; 4 Present Address: Strube research GmbH & Co. KG, Hauptstrasse 1, 38387 Söllingen, Germany; 5 Present Address: Bayer Crop Science, Alfred-Nobel-Str. 50, 40789 Monheim, Germany; 6 Present Address: Institute of Biochemistry and Molecular Biology I, Medical Faculty, Heinrich-Heine University, Düsseldorf, Germany; 7 Present Address: Genome Informatics Facility, Office of Biotechnology, 448 Bessey Hall, Iowa State University, Ames, USA

**Keywords:** High-throughput screening, Transcriptomics

## Abstract

Beet cyst nematodes depend on a set of secretory proteins (effectors) for the induction and maintenance of their syncytial feeding sites in plant roots. In order to understand the relationship between the beet cyst nematode *H. schachtii* and its host, identification of *H. schachtii* effectors is crucial and to this end, we sequenced a whole animal pre-infective J2-stage transcriptome in addition to pre- and post-infective J2 gland cell transcriptome using Next Generation Sequencing (NGS) and identified a subset of sequences representing putative effectors. Comparison between the transcriptome of *H. schachtii* and previously reported related cyst nematodes and root-knot nematodes revealed a subset of esophageal gland related sequences and putative effectors in common across the tested species. Structural and functional annotation of *H. schachtii* transcriptome led to the identification of nearly 200 putative effectors. Six putative effector expressions were quantified using qPCR and three of them were functionally analyzed using RNAi. Phenotyping of the RNAi nematodes indicated that all tested genes decrease the level of nematodes pathogenicity and/or the average female size, thereby regulating cyst nematode parasitism. These discoveries contribute to further understanding of the cyst nematode parasitism.

## Introduction

Beet cyst nematode *Heterodera schachtii* causes severe economic losses on several crops^1,2^. Due to the obligatory sedentary endo-parasitic life style, *H. schachtii* depends on a single hyperactive feeding site called syncytium during parasitism. Infective juveniles (J2) invade plant roots, migrate intracellular until they find the initial feeding cell which is later modified to serve as a feeding structure with the aid of secretions released from the esophageal glands^3^. Syncytium is induced by the fusion of several hundred cells after cell wall dissolutions along the plasmodesmata (PD)^4^. Such adaptation in the cyst nematodes created the need to evolve highly specialized mechanisms facilitating root invasion, host defense suppression, syncytium establishment and maintenance^4,5^. Nematodes inject a cocktail of cell wall degrading enzymes in order to soften the plant tissue and to facilitate penetration along and through plant cell walls^6–8^. In order to, thoroughly, understand the parasitism of cyst nematodes, it is crucial to have adequate knowledge about their effectors and their functions. Therefore, it is imperative to sequence the transcriptome of *H. schachtii* and identify effectors.

Expressed sequence tags (ESTs) of parasitic stage of multiple plant parasitic nematodes (PPNs) are available and serve as a useful source for effectors mining^9^. Microaspiration and sequencing of *H. glycines* gland cells excised from a range of parasitic stages detected 51 putative effectors^10^. A putative *H. schachtii* parasitome has been identified as 50 putative secretory proteins resulted from the ESTs^11^. A recent review showed that the number of available *H. schachtii* ESTs reached 2,182 sequences^12^. The use of the available EST sequences as a part of workflow determining excretory-secretory proteins aided in determining a dataset of putative secretory proteins of animal- and plant-parasitic nematodes^13^. One of the large resources of nematode transcripts is the NEMBASE4 database (http://www.nematodes.org/nembase4) containing ESTs from 324 different cDNA libraries from 63 different nematode species^9^. Though ESTs sequencing can be used to predict putative effectors, this method included a strong bias against the long transcripts and/or transcripts with a low expression. Also, as true N-terminal sequences are missing, that hinder the detection of signal peptides, this strategy can lead to false negatives. In contrast, Next Generation Sequencing (NGS) using Illumina provides unbiased fragmentation of RNA while sample preparation and a high sensitivity while transcript assembly.

Application of the NGS made it possible to sequence genomes of several PPN species including *Meloidogyne incognita*, *M. hapla*, and *Globodera rostochiensis*^1,14,15^. Such progress provides the possibility to perform comparative analyses to investigate aspects such as genome structure, regulatory sequences and alternative splicing, that were found to be important in *G. rostochiensis* parasitism^16^. Meanwhile, several *de novo* assemblies of PPN transcriptomes including that of *H. avenae*, *M. graminicola*, *Pratylnechus coffeae*^17–19^, have been recently published.

Sequencing of most PPN transcriptomes that aimed to identify putative effectors was mostly based on the bioinformatics categorization and filtering. To our knowledge, only a very few studies have utilized NGS to investigate transcripts expressed within the esophageal glands of PPNs such as *H. glycines*^10^ and *M. incognita*^20^

To gain insights into the *H. schachtii* transcriptome, we generated RNAseq libraries of *H. schachtii* whole pre-parasitic J2 worms, and esophageal gland cell of J2s and juveniles isolated after 5 days post inoculation. RNAseq libraries were sequenced using Illumina Paired-End sequencing technique, thus enhancing sequencing depth assembly and, at the same time, reaching a high sensitivity to detect transcripts that are expressed at a very low level. In this way, we predicted 178 putative effectors in *H. schachtii*. Our prediction procedures based on the RNAseq using a whole worm as well as esophageal glands of *H. schachtii* provides an innovative way to identify key effectors involved in cyst nematode parasitism in pre-infective and early parasitic (5 dpi) stages. Also, this study included experimental validation of 6 putative effectors using qPCR and *in situ* hybridization analyses. Knocking down three of the 6 putative effectors using RNAi has dramatically inhibited *H. schachtii* parasitism.

## Results

### Sequencing results and data processing

**Figure 1:**
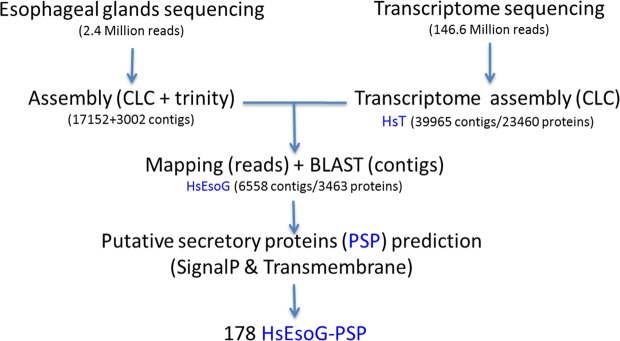
The pipeline to predict the *H. schachtii* esophageal gland related putative secretory proteins (HsEsoG-PSP).

**Figure 2:**
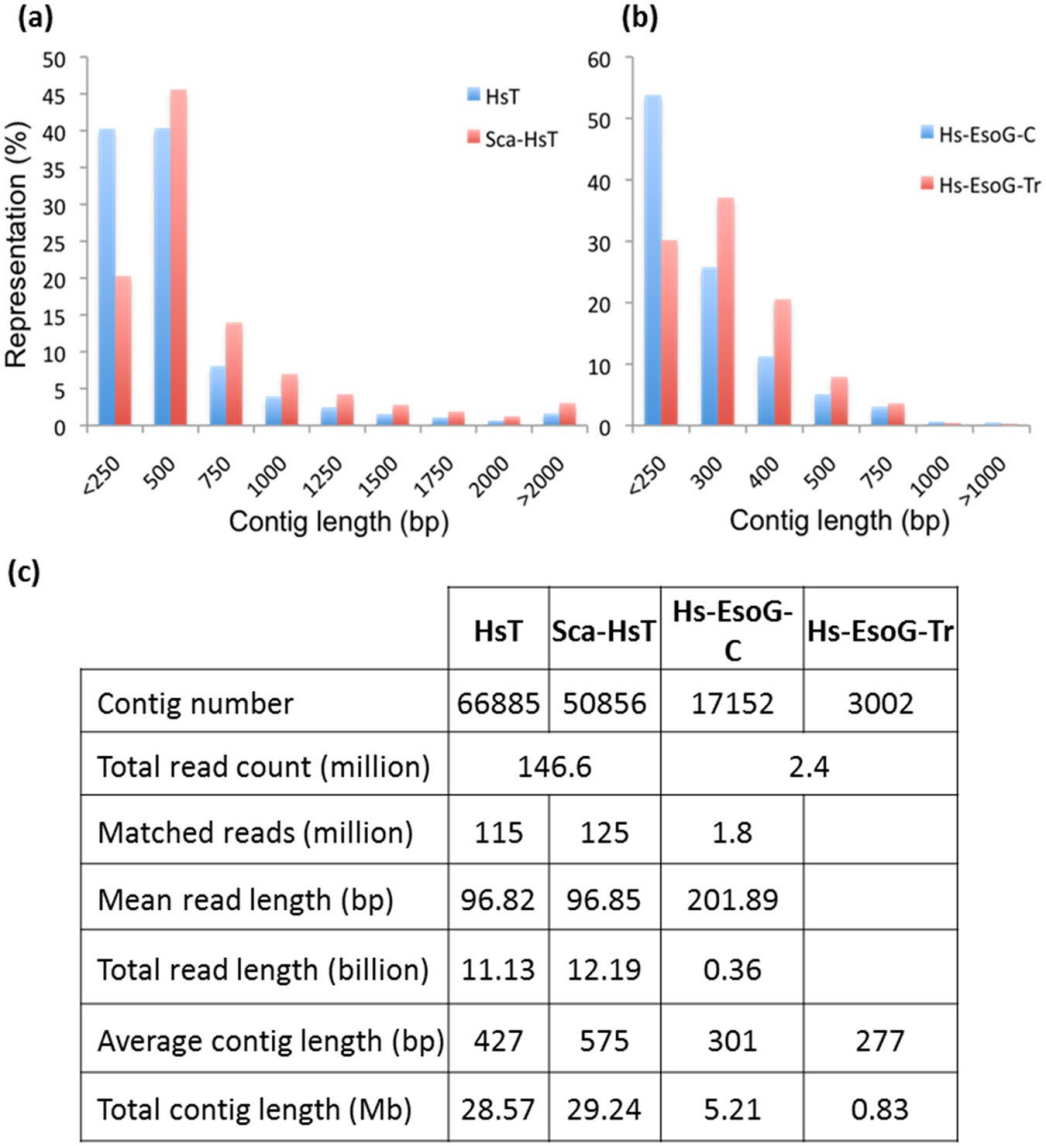
Reads and assembly details of *H. scahchtii* transcriptome. (**a**) Contig size distribution of *H. schachtii* transcriptome assemblies with scaffolding (ScaHsT) and without scaffolding (HsT). (**b**) Contig size distribution of *H. schachtii* esophageal gland transcriptome assemblies using CLC (Hs-EsoG-CLC) or Trinitiy (Hs-EsoG-Tr). (**c**) The details about *H. schachtii* transcriptome assemblies.

We sequenced and analyzed the transcriptomes of whole J2s and the esophageal gland cells of pre-infective J2 and post-infective J2 (5 dpi) (Fig. 1) parasitic stages to identify putative effectors. Sequencing of pre-infective J2 mRNA resulted in approximately 148 million reads (100 bp paired-end reads). Trimming for quality, ambiguity and adapter sequences followed by duplicate removal resulted in approximately 115 million reads. The trimmed reads were assembled without scaffolding, which resulted in a *de novo* transcriptome assembly of *H. schachtii* (HsT), which contained 66,885 contigs (data is available under NCBI project number PRJNA530532). All contigs added up to a total size of 28.6 Mbp with a GC content of 47%. A scaffold assembly (ScaHsT) was performed, which resulted in 50856 contigs with an average contig length of 575 bp. The ScaHsT contigs were useful in correcting the individual sequences by comparing them to HsT. A total of three sequencing runs, two runs J2 and one run 5 dpi, of esophageal gland-RNA resulted in a sum of 2.53 million reads with an average read length of 180 bp (a total of 437.6 Mbp) that were, subsequently, trimmed and it ended up to 2.49 million reads. The trimmed reads from each esophageal gland sequencing run that were combined and assembled once by CLC and once by Trinity, resulted in 17424 or 3002 contigs, respectively (Fig. 2). In order to relate the esophageal gland sequences to the Hs transcriptome we used 2 strategies: (1) mapping trimmed EsoG reads to HsT and (2) BLASTn of the HsT contigs against the assembled esophageal gland contigs (20426 contigs). (1) Mapping approach resulted in 5074 esophageal gland contigs after filtering out false positives excluded contigs with long (>50 bp) repeats and short contigs (<250 bp). (2) BLASTn of 50856 Hs contigs with the 5074 contigs of esophageal gland assemblies resulted in 4414 contigs. Merging both subsets to form *H. schachtii* gland transcriptome resulted in HsEsoG dataset with a total of 6558 contigs (Table S1).

### Comparison of HsEsoG with other sedentary nematode transcriptomes

**Figure 3:**
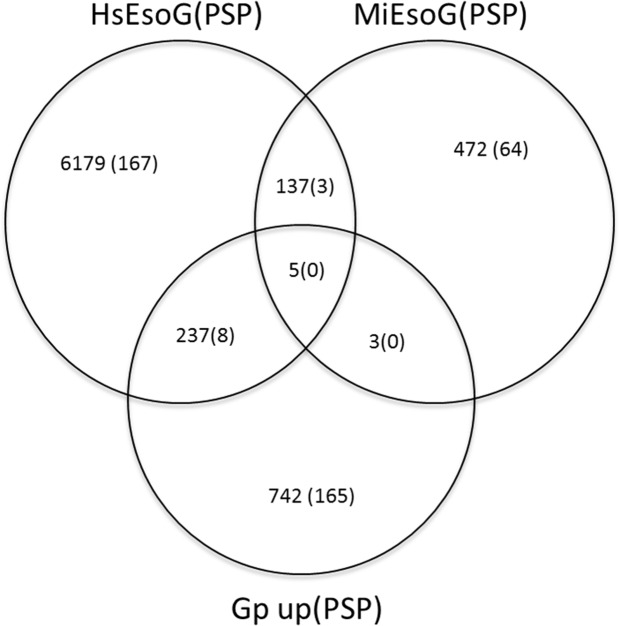
Venn diagram depicting the overlapping esophageal gland related sequences among *H. schachtii*, *M. incognita* and *G. pallida*. Numbers are representing protein sequences and numbers in parenthesis are the number of putative secretory (PSP) sequences.

To investigate the possibility of overlapping sequences between the 6558 HsEsoG-PSP subset and other available sedentary-nematode sequences. Sequences of *G. pallida*, *M. incognita* and *G. pallida* proteins that were upregulated in one or more developmental stages (Gp-up)^21^ were compared with the HsT contigs. A total of 987 homologous sequences were observed, which included 242 HsEsoG contigs. We identified 8 HsEsoG-PSP as a subset of the 242 HsEsoG contigs. Additionally, comparison of 3758 *M. incognita* proteins in esophageal glands^20^ with HsT contigs identified 617 sequences (142 sequences overlapped with HsEsoG and 3 of them were identified as HsEsoG-PSP) as shown in Fig. 3.

To determine whether our esophageal gland (EsoG) assembly and our J2 transcriptome (HsT) would be useful for effector identification, we searched our transcriptomes with previously-identified, putative effector datasets from cyst nematodes *H. schachtii* (64 sequences) and *H. glycines* (81 sequences) (Table S2). The putative-effectors datasets from *H. schachtii* and *H. glycines* were compared with the esophageal gland (EsoG) assemblies and HsT assembly by BLAST. We also compared these putative datasets against the *H. schachtii* transcriptome that was previously sequenced by 454 sequencing technology (Hs454)^22^. The highest number of matching effector sequences was in our *H. schachtii* transcriptome (62 sequences, 97%). Fifty-four putative effector sequences (84%) were identified in the Hs454 transcriptome. There were 38 sequences (58%) that could be found in the esophageal gland libraries (Table S2). Overall, we found that our J2 transcriptome library contained the highest number of known effectors, highlighting the utility of our detailed transcriptome data for *H. schachtii* effector searches.

### Functional annotation and secretory protein prediction

Gene Ontology (GO) analysis of the HsT assembly using the BLAST2GO ended up with 18157 contigs with GO-terms (Table S3). Meanwhile, the BLAST comparison against the Swiss-Prot and TrEMBL database has resulted in annotating 16872 and 23590 sequences, respectively. Annotation was enhanced using Assignment of Human Readable Descriptions (AHRD) tool (github.com/groupschoof/AHRD) which resulted in 23655 annotated contigs (Table S3). Translation of the longest open reading frames (ORFs) of *de novo* contigs (HsT) resulted in 23460 amino acid sequences. The predicted HsT protein sequences were analyzed for the presence of signal peptide and lack of transmembrane domain. Out of the total tested sequences, 1081 putative secretory proteins (HsPSP) were identified.

**Figure 4:**
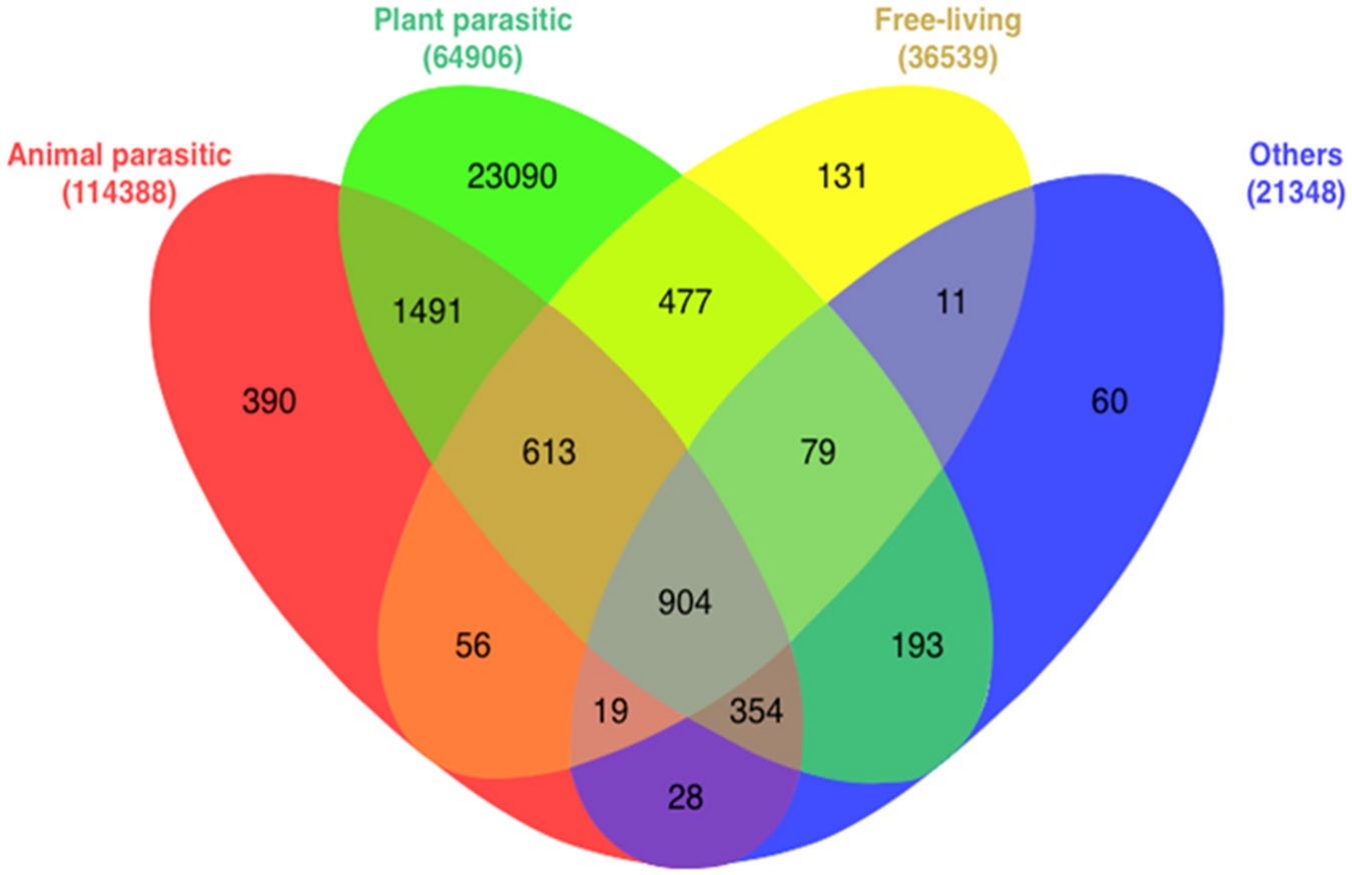
A Venn diagram showing the BLAST results of *H. schachtii* transcriptome assembly against Nembase4 ESTs database. All ESTs were categorized based on the feeding habits of their source nematode species into: animal parasitic (red), plant parasitic, (green); free-living (yellow); and others, which represent the rest of the species (blue). The numbers in parentheses represent the number of ESTs in each category and the numbers within each category represent the number of *H. schachtii* contigs that have a positive hit within this category (bit score >50).

HsT assembly was compared to the Nembase4 database after categorizing its ESTs into PPN, Animal-Parasitic Nematodes (APNs), Free-Living Nematodes (FLN) and Entomo-Pathogenic Nematodes (EPN). This comparison resulted in identification of a PPN-specific subset that included 23090 contigs, which were exclusively similar to PPN ESTs. The PPN-specific subset included 484 PSP (P-PSP) as shown in Fig. 4. The annotation results using *C. elegans* CDs has revealed 375 contigs out of the 484 P-PSP, which updated the P-PSP to total 109 contigs (Table S3).

### Identification of novel effectors

We performed a BLASTn to test the presence of *H. schachtii* ESTs (1212 ESTs)^10^ among the HsT contigs. Out of 1212 sequences, 1155 ESTs showed positive results of bit score >100. Furthermore, we tested the potential of the HsT in extending some of the 1212 ESTs aiming to identify more putative secretory proteins. A comparison of the predicted PPN-specific contigs (23090 contigs) determined by the Nembase4 BLAST against the 1212 sequences that were assembled from *H. schachtii*^11^ was performed and this resulted in 1421 contigs that had hits within the dataset (bit score >50, evalue <e-10, and hit length >100 bp). Further analysis of these contigs showed that out of the 1421 contigs, 24 proteins are determined as P-PSP and were not described among the previously published effector dataset. Four out of the 24 contigs showed a high similarity with the *G. pallida* cDNA and were significantly similar to *H. glycines* sequences. Amplification and sequencing of those contigs showed full match to the *H. schachtii* assembly. Among the identified 24 contigs, Contig_12694 (*Hs-tyr*)^23^, which is one of the HsEsoG-PSP and one of the 8 sequences, which overlap with the MiEsoG-PSP (Table S3).

Furthermore, the presence of most, if not all of the known *Heterodera spp*. effectors within the HsT can represent its coverage level, determine homologous effectors and differentiate the novel effectors within the whole HsPSP. In order to test this point, we used a compiled, out of several publications, dataset of putative effector sequences from *H. schachtii* (64 sequences) and *H. glycines* (81 sequences) that were subjected to BLAST against the HsT assembly (e-value <e-10). As shown in Table S2, a large proportion of the putative secretory proteins and effectors were identified. Eighteen matching contigs were included within the HsEsoG (out of the 18 contigs, contig_44453^24^ and contig_16417 were HsEsoG-PSP and 16 contigs were not identified as HsPSP) as shown in Fig. 4.

**Figure 5:**
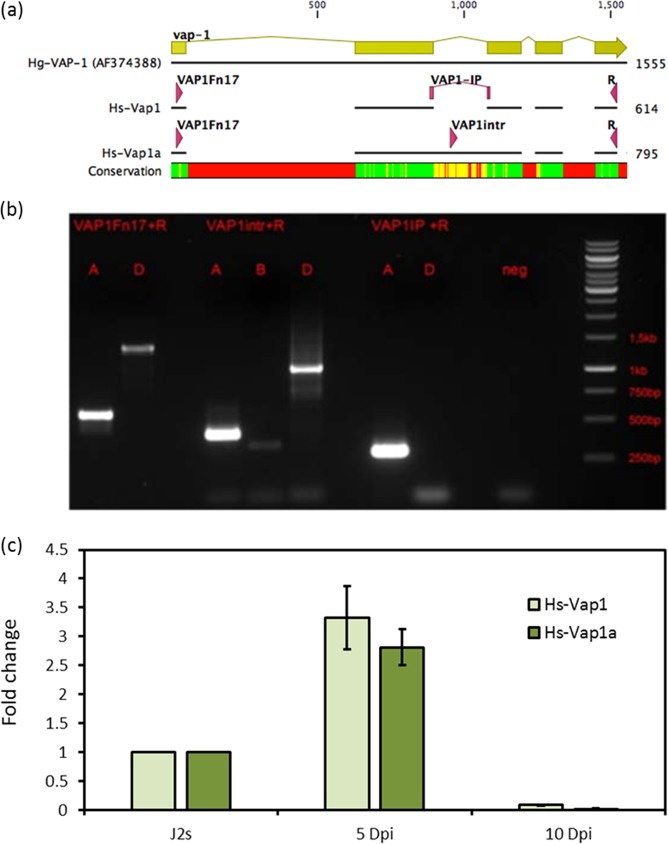
Alternative splice variants of Hs-vap1. (**a**) Alignment of *H. glycines* Vap1 (AF374388) gene and *H. schachtii* Vap1 transcript isoforms (Hs-Vap1 and Hs-Vap1a). (**b**) PCR amplification results. Lanes are grouped by the pairs of primers used for amplification (written on the top of each group) and each lane are marked as (A) lanes where cDNA was used or (D) lanes where DNA was used. N = negative. (**c**) Expression level of Hs-Vap1 isoforms in 5 and 10 dpi. The forward primers are mentioned above each lane. Lane M: 1Kb DNA marker.

Interestingly, the comparison between HsT and HgPSP led to discovery of an alternative spliced venom-allergin-like protein 1 (Hs-vap1a) transcript. The presence of both transcripts, Hs-vap1 and Hs-vap1a, was validated by generating cDNA, cloning and sequencing. To study their expression profile, each isoform was amplified using rtPCR and qRT-PCR of stage specific cDNA with specific primers (Table S4). Accumulation of both transcripts was highest in post infective J2 (5 dpi), and lowest in young females (10 dpi) (Fig. 5).

### Identification of HsEsoG-PSP

One of the strong evidences to consider PSP as a putative effector is to analyze expression of the PSP within the esophageal gland. Mapping of the esophageal gland reads to the HsT resulted in 5092 contigs that were successfully mapped. Furthermore, BLAST comparison of HsT against the esophageal gland assemblies resulted in identification of 4414 contigs. A total of 6558 contigs were identified as HsEsoG, which represent the result of merging the BLAST and the mapping results due to the presence of 2930 contigs (49.6%) as common contigs in both datasets. HsEsoG was found to include 178 PSPs and it was designated as HsEsoG-PSP (Table S1).

**Figure 6:**
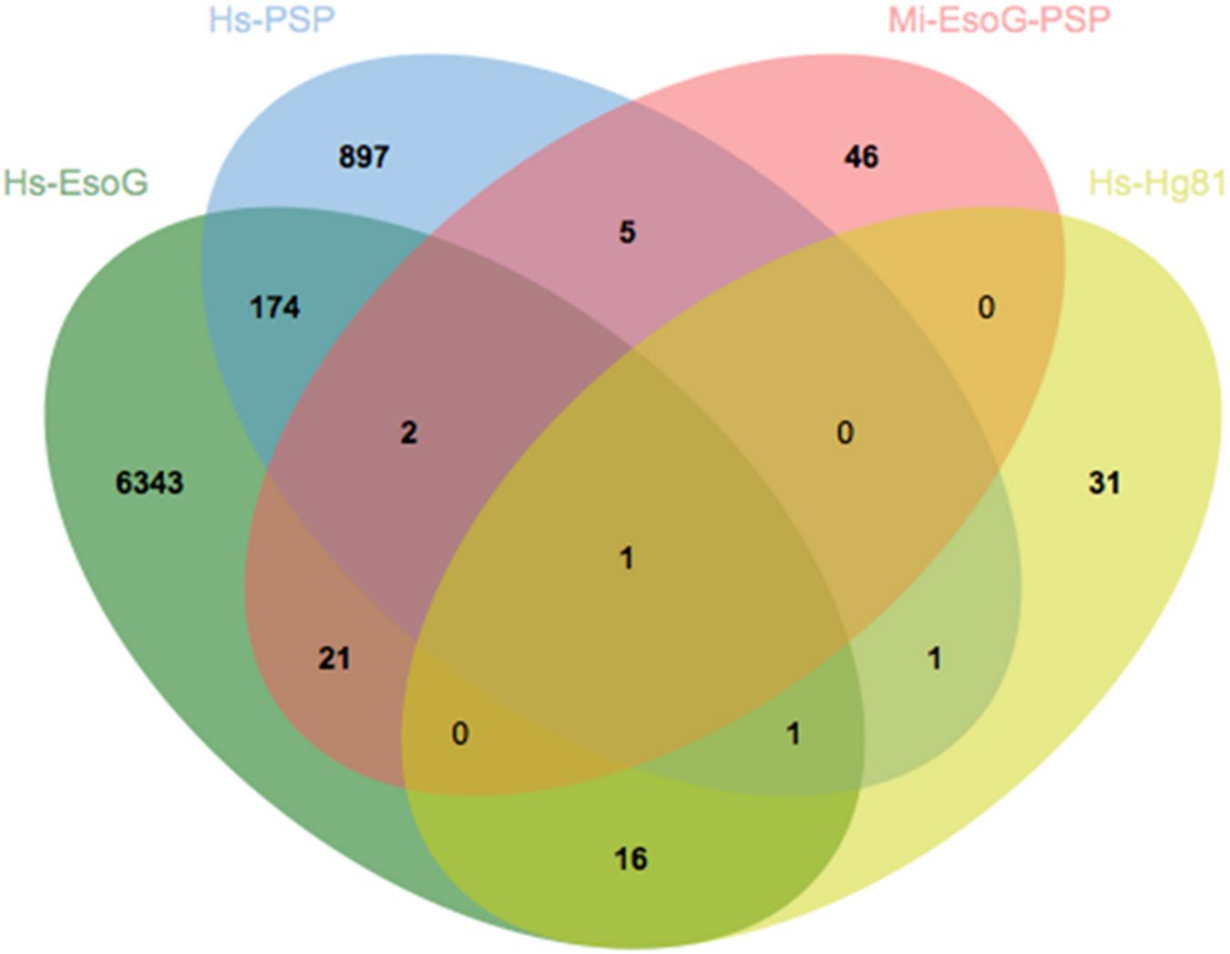
Venn diagram depicting the overlapping of *H. schachtii* sequences with other nematodes. The Venn diagram represents *H. schachtii* esophageal gland related sequences (HsEsoG) and *H. schachtii* putative secretory proteins (HsPSP) with homologous esophageal gland putative secretory proteins from *M. incognita* and *H. glycines*.

Furthermore, we used the previously identified *M. incognita* (MiEsoG) and *H. glycines* (HgEsoG) esophageal gland related sequences^10,20^ to test similarity with the HsEsoG and related PSPs. Among the 3758 MiEsoG genes, 617 genes were similar to the HsT and this included 142 contigs identified in HsEsoG. The identified 142 HsEsoG contigs included 3 HsEsoG-PSP. In comparison with the 81 *H. glycines* (HgEsoG) genes, which were identified as PSP 50 homologs, were identified within the HsT that included 18 HsEsoG contigs (2 HsEsoG-PSP). The comparison has resulted in the identification of 1 PSP common in all datasets and 2 PSPs common in HsEsoG and MiEsoG but not in Hg81 (Fig. 6).

### Gene set enrichment analysis (GSEA)

GSEA was used to identify the nature of genes of interest at a functional level. In this analysis, we tested the HsPSP, HsEsoG, HsEsoG-PSP, MiPSP, MiEsoG, MiEsoGPSP, MiEsoG-PSP, and *G. pallida* (GpPSP) data. Testing for common GO annotation among the HsPSP, MiPSP, and GpPSP datasets revealed the following GO terms: Carbohydrate binding, catalytic activity, lipid metabolic process and catalytic activity. Our results showed the presence of 28 GO terms overlap between HsEsoG and MiEsoG GSEA (Table S5). The GSEA test in the HsEsoG-PSP and MiEsoG-PSP detected 4 GO terms enriched (lipid transporter activity, lipid transport, serine-type endopeptidase inhibitor activity, and glycerol ether metabolic process) in the MiEsoG-PSP and no enrichment within the HsEsoG-PSP (Table S5).

### Validation and functional analysis

**Figure 7:**
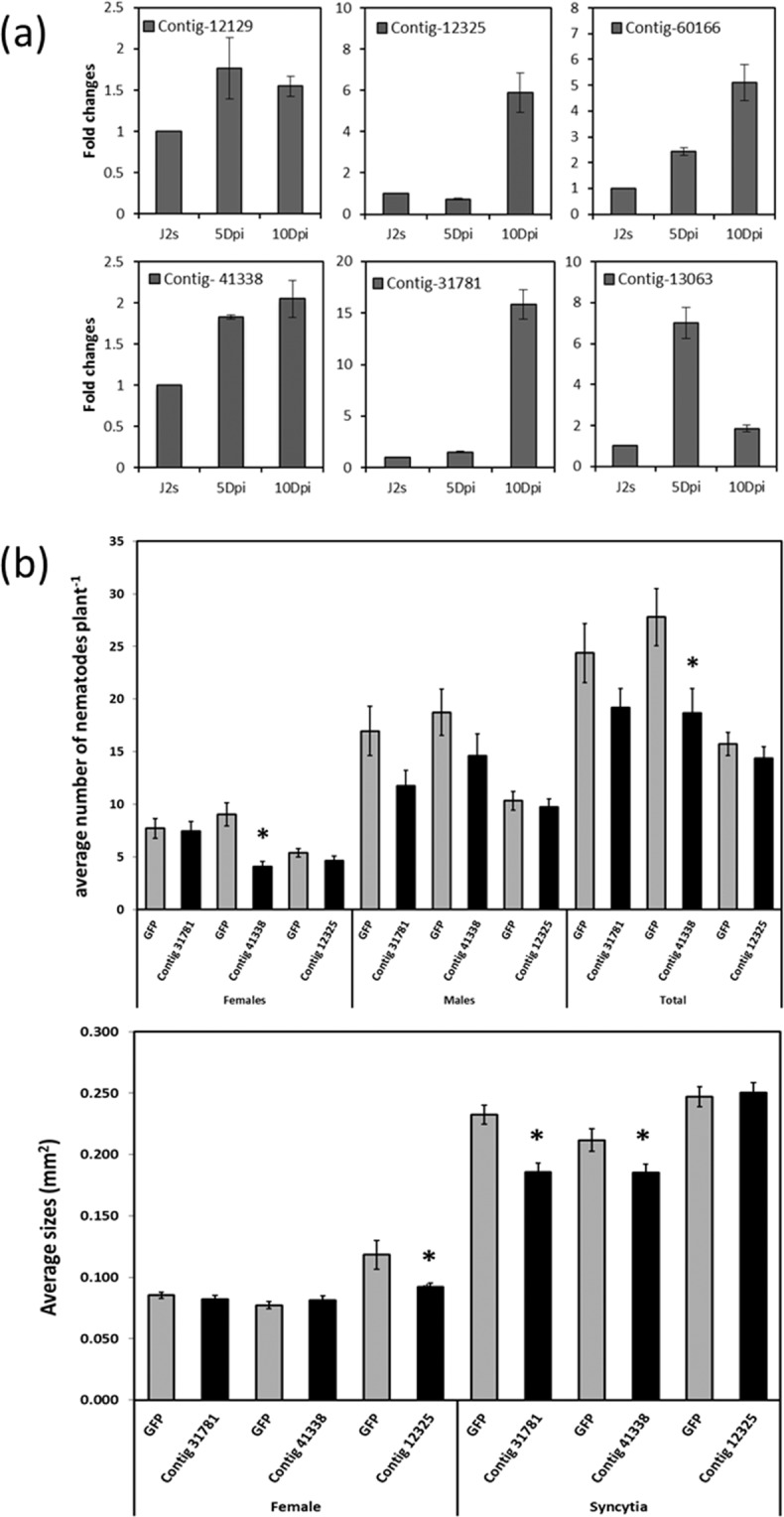
Experimental validation of a subset of Hs-EsoG-PSP. (**a**) qPCR, (**b**) RNAi, and each bar represents the mean ± standard error (n >35). Asterisk indicates significant differences based on Student’s *t*-test (P < 0.05).

A few of the identified HsEsoG-PSPs (contig-12129 (Nuclear hormone receptor), contig-12325 (Exostosin-1), contig-60166 (Probable replication factor), contig-41338 (hydroxyglycine alpha-amidating lyase), contig-31781 (farnesyltransferase), and contig-13063 (putative esophageal gland cell secretory: *msp2*^15^)) were randomly selected for experimental validation. The selected sequences were experimentally validated by profiling their expression level throughout the nematode development by qPCR analysis. Expression of the selected sequences was upregulated in the early- (5 dpi) and/or late-parasitic (10 dpi) stages (Fig. 7a). Furthermore, three sequences were selected out of the 6 tested sequences in Fig. 7a to be knocked down in J2s using sequence-specific dsRNA. The results of knocking down these sequences showed a significant inhibition of nematode parasitism. Knocking down contig 41138 has resulted in a significantly less total number of infecting nematodes, which has mainly resulted of a significant lower number or females and a statistically non-significant lower number of males. RNAi of contig_12325 revealed smaller females, while RNAi of (contig_31781 and contig_41338) resulted in a reduction in the size of syncytia (Fig. 7b).

## Discussion

Analyzing the transcriptome sequences in order to identify the secretome of plant parasitic nematode species is a valuable source to investigate the nature of the biological processes associated with this type of parasitism. A successful identification of the effector proteins would depend on a reliable identification of the putative secretory proteins. Reaching that aim is facing certain challenges, most importantly the source of the used materials, which developmental stage, organ, and/or the purity of the used materials. Meanwhile, the level of previous knowledge about sequences from other nematode species is playing a key role ending up to optimal annotation. In this study, we sequenced RNA from *H. schachtii* J2 that were cultured *in-vitro*, and eggs were hatched under sterile conditions. The juveniles that were used were hatched without any contact with plant tissues or plant exudates. These conditions assured us that resulted sequences would be only a result of sequencing *H. schachtii* RNA excluding RNA traces from the plant host or any microbial contamination. Using next generation sequencing, Illumina Paired-end sequencing technique was selected to enhance the assembly and reaching a high sensitivity for detecting transcripts that would have a low level of expression, which would fit to the expectation about an effector expressed within J2 as a pre-infective stage.

In our study, we sequenced and analyzed transcriptomes of whole pre-parasitic J2s and transcriptomes of esophageal glands of pre-parasitic and parasitic J2 of *H. schachtii*. By *de novo* transcriptome assembly, we presented three types of *H. schachtii* secretomes. Identification of putative effectors in HsT in comparison with the published putative effectors of *H. schachtii* and *H. glycines* indicated that the HsT represents significantly higher number of novel Hs putative effectors. Nevertheless, it is worth mentioning that the relatively low number of esophageal gland reads may not be capable to capture the complete gland transcriptome, especially, the low expressed transcripts, which may have led to miss some novel effectors.

Processing of HsT resulted in the translation of 23460 contigs based on the predicted longest ORF. Prediction of signal peptides and transmembrane domains resulted in the identification of 1081 putative secretory proteins (PSP), which represent 4.6% of the total protein dataset (23460 proteins). In order to have an impression of the expected range of PSP sequences within the root-knot nematode *M. incognita* and cyst nematode *G. pallida*, we analyzed their protein datasets for signal peptide and transmembrane domains and identified 1505 proteins (7.4% out of 20485 proteins) and 1592 proteins (7.5% out of 21232 proteins), respectively. Meanwhile, this data matches the result of a previous study using the ESTs that aimed to discover putative excretory-secretory proteins where the authors described a range of 2.7–4.4% of putative secretory proteins within the cyst- and root-knot nematodes^13^. The low number of PSP within our dataset could be a result of the partial sequences or missing PSPs that are exclusively expressed within developmental stages other than J2.

On the other hand, the HsT includes isoforms of transcripts (i.e. VAP1 alternative spliced form (Fig. 5)), which caused an increase in the total number of the assembled contigs in contrast to *M. incognita* and *G. pallida*. Comparison of the HsT assembly with the previously known effectors of *H. schachtii* and *H. glycines* showed that the sequencing of *H. schachtii* pre-parasitic J2 using Illumina sequencing lead to identification of nearly all known PSPs of *H. schachtii* and *H. glycines*. This apparently indicates the sensitivity of the NGS that can lead to sequence transcripts that have a very low level of expression. Furthermore, only 2 transcript out of the previously published Hs-PSPs^11^, which did not match the HsT (gi|32325259 and CL255Contig1) did not match with any of the available sequences within the NCBI. We assumed that this resulted from misassembled ESTs.

This study has made it possible to sequence and investigate *H. schachtii* transcriptome. Also, it facilitated the transcriptome comparison among HsT, *G. pallida* and *M. incognita*. The ability to identify a subset of putative secretory proteins that are related to the esophageal gland in a high throughput manner is an added value to the *in-silico* selection processes. The fewer number of the overlapping PSPs between *M. incognita* and *H. schachtii* (142 PSPs) than the overlapping PSPs between *G. pallida* and *H. schachtii* (242 PSPs) may suggest a more common PSPs functions between cyst nematodes comparing with the PSPs functions between cyst- and root knot-nematodes. However, this finding is logically expected as it is successfully reflecting the higher evolutionary distance in the first comparison compared to the latter, it needs to be confirmed by using more species representing each group.

In all, we identified 178 novel putative Hs effectors by a comparison between our Hs esophageal gland related sequences and known esophageal gland sequences^10,20^. As a result of comparison with the identified putative effector from *M. incognita* (Mi-EsoG-PSP) and *H. glycines* putative effectors, 4 out of the 178 putative effectors had an overlap, contig_12189, contig_12694, contig_44453, and contig_16417. Contig_12694 is *Hs-Tyr* (KU975565.1), which was annotated as tyrosinase^23^ overlapped with Mi-EsoG-PSP. Contig_44453 is *Hs-PDI* (KU948160.1), which was annotated as protein disulfide isomerase^24^ overlapped with both Mi-EsoG-PSP and *H. glycines* putative effectors.

While our transcriptome analysis aimed to enhance our knowledge about the transcriptome of *H. schachtii*, it enabled us to discover putative secretory proteins. We used several approaches to identify new putative effectors such as comparison of our *H. schachtii* transcriptome assembly with the ESTs of *H. schachtii* and other related PPNs. Earlier, identification of 50 proteins in the *H. schachtii* parasitome EST study resulted in 1212 transcripts^11^. We anticipated that the deep sequencing by Illumina could extend the earlier reported partial ESTs^11^ and would make it possible to identify complete cDNA. We compared the 1212 sequences with the 23090 contigs that exclusively were BLAST hits within the PPN ESTs in NEMBASE4. We expected that a subset with these features would be enriched with proteins related to parasitism. By this approach, we characterized 24 proteins (Table S3) within the HsT that were not included within the previously published secretome^11^. One of these contigs is the published *Hs-Tyr* (contig-12694) that was shown to be important for parasitism and involved in modulating plant hormones homeostasis which is supported by several experimental evidences^23^. These results are a prove of concept and show the potential of such approach and the data from HsT to identify effector proteins. Additionally, comparison of our HsT assembly to *H. glycines* gland-proteins resulted in the identification of 24 homologous sequences that were not described within the *H. schachtii* secretome (Table S3).

Functional enrichment within the identified putative Hs-PSP, EsoG-PSP, and EsoG-PSP showed no big overlap among the PSP datasets of *H. schachtii*, *M. incognita* and *G. pallida*, while the level of enrichment and enrichment overlaps has clearly increased between the esophageal gland datasets of *H. schachtii* and *M. incognita*. These results suggest that the functional activities of the esophageal glands of both species are basically more conserved matching the basic biological needs in contrast to their PSPs, which evolved differently to match the parasitizing needs for each species.

The consistent upregulation, within at least one of the post infective stages, of all selected putative effectors has confirmed that these proteins would play a role within the nematode post parasitic stages. Knocking down 3 of the 6 selected putative effectors has shown that two out of the three putative effectors have caused a significant negative effect on parasitism by affecting the size of the nematodes and/or the size of the syncytia.

Application of NGS of the whole transcriptome and genome represents an effective way to further expand our knowledge about the PPNs at the molecular level^19,21^. The ability of deep sequencing leads to identification of nematode pathogenicity related transcripts, which would lead to develop strategies in the nematode management. A successful nematode management program can benefit from accurate nematode identification using sequence based molecular markers on PPN species and using nematode resistant cultivars based on identification of effectors, which can lead to the identification of their host-target genes to facilitate knowledge-based plant breeding programs^25^.

## Methods

### Nematode cultures

Mature cysts of *H. schachtii* were collected from white mustard (Sinapis alba L.) cvar. Albatros plants in funnels and hatched in 3 mM ZnCl_2_^26^. Freshly hatched pre-parasitic second stage juveniles (J2s) were collected for RNA extraction, while, the rest of J2s were used for infecting the Arabidopsis plants for post infective stages collection and for infection assay. For the esophageal gland work, mixed parasitic stages of the nematodes were isolated by macerating infected roots in a blender followed by sieving and separation on a sucrose gradient^27^ and gland cells were isolated.

### Plant-parasitic nematode gland cell isolation

Parasitic nematodes were collected from infected plants and suspended in 100 ul Phosphate Buffered Saline (pH 7.4) and mixed with SUPERase-In (Life Technologies, Grand Island, NY, U.S.A.) (1 U/μl). The nematode suspension was transferred into a 60 mm baked Pyrex petri dish pretreated with RNaseZap (ThermoFisher, Waltham, MA, U.S.A.) and cut with a sterile razor blade on a benchtop vortex (Vortex Genie 2, ThermoFisher, Waltham, MA, U.S.A.). Cutting progress was monitored with a dissecting microscope, and the cut nematodes were washed into RNA-grade 100% ethanol and stored at −80 °C. Nematode homogenates were fixed in ethanol for at least 24 hours prior to use and centrifuged in a swing bucket rotor for 3 minutes at 600xg and to the pellet, 100 μl of HistoGene stain (Applied Biosystems, Carlsbad, CA, U.S.A.) supplemented with 1 U/μl Superase-was added. After staining the nematode tissues for 1 min at room temperature, staining was stopped by adding 100% ethanol. The stained tissue was centrifuged for 1 min at 600xg and supernatant was removed. The pellet was washed once with 100% ethanol and resuspended in 300 μl Halocarbon Oil 700 (Sigma-Aldrich, St. Louis, MO, U.S.A.). The tissues were then transferred onto a glass slide of coverslip thickness previously treated with RNaseZap and mounted onto a Zeiss Axiovert 100 (Carl Zeiss, Thornwood, NY, U.S.A.) inverted compound microscope equipped with a glass needle holder attached to a micromanipulator, in line with a Cell Tram (Eppendorf, Hauppauge, NY, U.S.A.) oil filled piston. A borosilicate glass needle (pore size 20–30 μm) was inserted into the needle holder. The Cell Tram was used to generate a negative pressure on the needle to isolate individual gland cells from the halocarbon oil. Collected single-cells of each nematode species were pooled and stored at −80 °C until RNA isolation.

### RNA isolation

For whole nematode transcriptome work, pre-parasitic J2swere used. Total RNA was extracted using mirVana total RNA extraction kit (Applied biosystems) by following the manufacturer instructions. Quality and quantity of the extracted RNA were tested using the Agilent 2100 Bioanalyzer system (Agilent Technologies). Two independent RNA samples, with RIN (RNA Integrity Number) value greater than 9.0, were used independently for 100 paired-end sequencing. For esophageal gland RNA-seq, gland cells in stored halocarbon oil were mixed with mineral oil (Sigma-Aldrich) at a 1:5 ratio and centrifuged for 1 min at 10,600xg. After confirming no separation of oil layers, total RNA of the gland cells was extracted using the Arcturus PicoPure RNA Isolation Kit (Applied biosystems) with slight modifications. To the oil mixture containing gland cells, equal volume of extraction buffer was added, thoroughly mixed and incubated at 42 °C for 30 min. The sample was then centrifuged for 1 min at 10, 600 g and lower extraction buffer phase was collected. Cellular debris were removed by centrifugation for 2 min at 3,000xg and total RNA was isolated according to the manufacturer’s instructions and stored at −80 °C until cDNA library construction.

### RNA-seq analysis

For whole transcriptome work, mRNA was fragmented using divalent cations under elevated temperature. cDNA was prepared for each run separately and fragments with an average length of 330 bp were used for further processing. For gland cell specific transcriptome, total RNA of gland cell was subjected to rRNA depletion using the Ribo-zero Magentic Kit (Human/mouse/rat) (Epicenter), reverse transcribed and cDNA was amplified using the SMARTER Stranded RNA-seq kit (Clontech) according to the manufacturer’s instructions. Sequence libraries were prepared using the Illumina Indexing Primer Set and the barcoded libraries were quantified using a Qubit Fluormeter (Thermo Scientific Life Technologies). Sequencing of the pooled libraries was carried out on an Illumina MiSeq sequencer with 250 base reads (2.4 million reads).

### Trimming and assembly of Illumina sequence reads

Sequences trimming, duplicates removal and transcript assembly was performed with the CLC genomic workbench (version 5.1). Trimming for quality was performed excluding low-quality bases (>0.05), ambiguous nucleotides (>2n) and adapter sequences were also removed. The *de novo* assembly of all reads that passed quality filtering was computed with following parameters: word size 24, similarity = 0.8, length fraction = 0.5, insertion cost = 3, deletion cost = 2, and mismatch = 2. In this study, we performed two transcriptome assemblies, one assembly that included scaffolding and another assembly without scaffolding. All contigs were translated based on their longest ORF and the orientation of their best hit using BLAST2GO and EMBOSS Transeq tools.

### Functional description and annotation

All contigs were annotated by using Swiss-Prot (BBH), TrEMBL (BBH), and *C. elegans* (BBH)^28^ and the results were used as an input for the Automated Human Readable tool (AHRD). Furthermore, BLAST2GO (version 2.6.0) was used to assign gene ontology (GO) terms and for Gene Set Enrichment Analysis (GSEA). Functional annotation was done for the *H. schachtii* contigs and the protein data sets of *M. incognita* (http://www.inra.fr/meloidogyne_incognita). The nr-database was used for the blast search with an e-value threshold of 1e-6. GO-terms were assigned with an annotation cutoff of 55 and a GO-term weight of 5. The HsEsoG and MiEsoG subsets representing the esophageal gland-related genes of *H. schachtii* and *M. incognita*, respectively, were analyzed by *Fisher Exact* test to perform a Gene Set Enrichment Analysis GSEA. All contigs were BLAST compared.

### Developmental expression pattern analysis

Transcription level of 6 contigs was analysed in different developmental stages including pre-parasitic J2, parasitic juveniles and females by quantitative PCR (qRT-PCR) using specific primers (Table S4). Around 3000 pre-paparsitic J2s were collected directly from funnels^26^. Around 500–600 parasitic nematodes were collected manually by separating them from the *A. thaliana* roots after 5 and 10 dpi representing J3s and young females, respectively.

Expression analysis was performed as presented previously^23^. Total RNA was extracted using NucleoSpin RNA kit (MACHEREY-NAGEL) following the provided protocol. RNA quality and quantity were tested using the Agilent 2100 Bioanalyzer system (Agilent Technologies). First strand cDNA was synthesized using High-Capacity cDNA Reverse Transcription Kit (Applied Biosystems) in the presence of the oligo(dT) primer. The resulted cDNA was tested for the expression changes using the Stepone Plus Real-Time PCR System (Applied Biosystems) following the setups published in previous study^23^. The data were analysed using one-step software system to create Ct values. The results were analysed and relative expression was calculated following Pfaffl^29^. Actin was used as an internal control for all experiments. Three biological replicates from each developmental stage and three technical replicates for each sample were used.

### Double-stranded RNA (dsRNA) and gene silencing in nematodes

The dsRNA of three contigs (contig_31781, contig_41338 and contig_12325) was synthesised using MEGAscript T7 kit (Ambion, Life Technologies) following the manufacturer’s instructions. The *GFP* template was used for synthesis of a dsRNA construct that was used as a negative control. Freshly hatched J2s of *H. schachtii* were soaked overnight in 50 µL soaking mix (25 µL dsRNA 1 µg/µL, 5 µL soaking buffer 10×, 1.5 µL 100 mM spermidine, 5 µL 500 mM octopamine, 13.5 µL nematodes). Incubated J2s were washed with sterile water, then sterilized in 0.05 M HgCL_2_ for 2 minutes and washed again three times with sterile water. After sterilization, nematodes were divided into two sets. One set was used for analysis of expression of target genes using qRT-PCR as described above and the other part was used for the infection assay. Ten days old plants cultured on 0.2% modified Knop medium were inoculated with 60–70 J2 nematodes/plant. For each experiment 20 plants/treatment were used.

Each contig was tested separately from the other contigs by performing 3 experiments where dsRNA of the selected contig was used to knock down that contig and other plants were treated with GFP dsRNA as a negative control. Number of male and female nematodes per plant was counted after 12 days after inoculation (DAI). Furthermore, average size of female nematodes and associated syncytia were measured at 13 DAI using Leica M165C Binocular (Leica Microsystems, Wetzlar, Germany) and Leica Application Suite software. Experiment was repeated three times and statically analysed using the Student’s *t*-test.

### Ethical approval

All experimental protocols were approved by University of Bonn Or by Iowa state university (depending on the institute where the experiments were done).

## Supplementary information

Table S1.

Table S2.

Table S3.

Table S4.

Table S5.

## Data Availability

The data described in this manuscript is available under NCBI (project number PRJNA530532).
